# Imaging findings of primary hepatic leiomyosarcoma: a case report and literature review

**DOI:** 10.3389/fonc.2024.1490717

**Published:** 2024-11-21

**Authors:** Lifen Yan, Runqian Huang, Shuting Chen, Jiawei Chen, Jinglei Li

**Affiliations:** ^1^ Department of Radiology, Guangdong Provincial People′s Hospital (Guangdong Academy of Medical Sciences), Southern Medical University, Guangzhou, China; ^2^ Department of Radiology, People’s Hospital of Yingde City, Yingde, Guangdong, China; ^3^ Department of Radiology, Key Laboratory of Molecular Diagnosis & Disease Prevention, Heyuan People's Hospital, Heyuan, China

**Keywords:** liver, leiomyosarcoma, diagnostic imaging, tomography (X-ray computed), magnetic resonance imaging

## Abstract

Primary hepatic leiomyosarcoma (PHLS) is an extremely rare malignant tumor, which is often elusive in early diagnosis due to its rarity and nonspecific clinical and imaging presentations. Herein, we present a case of PHLS in a 66-year-old male and a review of the English literature from January 2000 to December 2023, focusing on the clinical and imaging characteristics of 30 patients with PHLS. The present patient was admitted to our hospital with complaints of abdominal distension, with history of hepatitis B. Tumor markers, including alpha-fetoprotein, carcinoembryonic antigen, and CA 19-9, were within the normal range. A hepatic tumor was incidentally identified during an abdominal ultrasound examination, further evaluated by contrast-enhanced CT and MR scans, which was preliminarily misdiagnosed as hepatocellular carcinoma. The tumor was surgically excised and definitively diagnosed as PHLS, characterized by two distinct areas with varying imaging features on contrast-enhanced CT and MR images. PHLS typically manifests as a well-defined, heterogeneously hypo- or iso-dense mass on CT, with a slightly prolonged T2 signal on MRI, and exhibits gradual enhancement during dynamic contrast-enhanced imaging. We advocate that the possibility of PHLS should be considered when the aforementioned imaging features are observed.

## 1 Introduction

Leiomyosarcoma is a malignant mesenchymal tumor originating from smooth muscle lineage (1), which most commonly arises in the uterus, retroperitoneum, soft tissues and the alimentary tract. In clinic, primary hepatic leiomyosarcoma (PHLS) is extremely rare, which is difficult to make accurate pre-surgical diagnosis, due to its rarity, nonspecific clinical and imaging manifestations, and lack of recognition. In this study, we present a case of PHLS that was pathologically confirmed following surgical resection, and provides a systematic review of PHLS in the English literature for comparison, focusing on its clinical characteristics and imaging findings.

## 2 Case description

**Figure 1:**
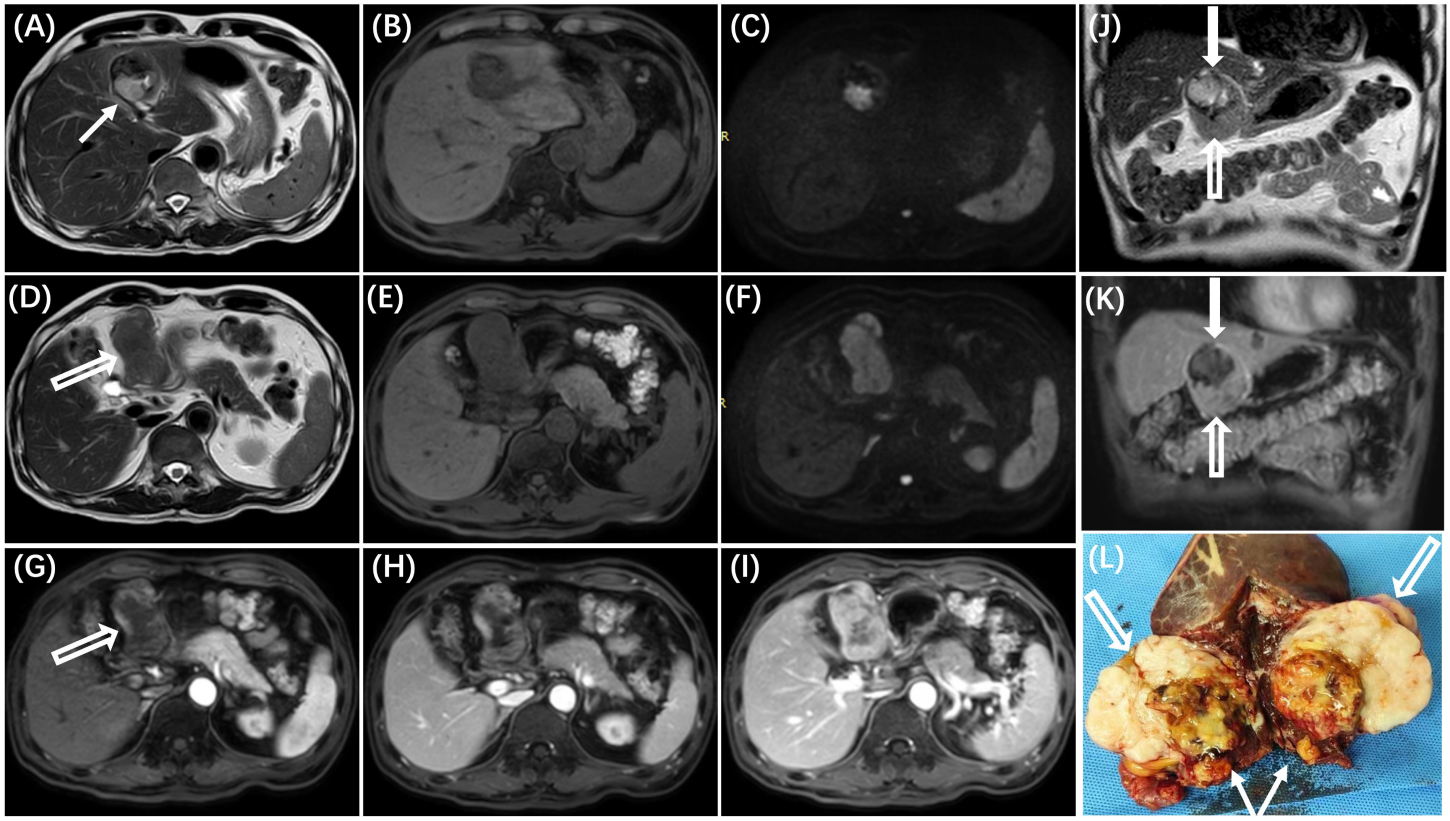
MR imaging reveals two distinct areas within the tumor, each with varying imaging characteristics. The area adjacent to the liver (**A–C**, white solid arrow in **J**) demonstrates mixed high and low signals on T_2_WI **(A)** and T_1_WI **(B)**, corresponding to the undifferentiated region with necrosis and hemorrhage identified in pathological examination (white solid arrow in **L**). The area distant from the liver (**D–F**, white hollow arrow in **J**) exhibits mild hyperintensity on T_2_WI **(D)** and hypointensity on T_1_WI **(E)**, correlating with the differentiated region observed in pathological examination (white hollow arrow in **L**). On dynamic contrast-enhanced images, the tumor displays heterogeneous enhancement during the arterial phase **(G)** and gradual enhancement in the portal venous and delayed phases **(H, I)**, with different degrees of enhancement observed in the two areas (solid arrow and hollow arrow in the **K**). On diffusion-weighted imaging (DWI), the mass (white arrow) shows hyperintensity **(C, F)**.

A 66-year-old man presented to a local hospital with a complaint of abdominal distension. An abdominal ultrasound examination revealed a large mass in the hepatogastric space. Subsequently, contrast-enhanced magnetic resonance (MR) imaging of the abdomen was performed (**Figure 1**) to better characterize the lesion. The mass demonstrated predominantly slight hyperintensity on T_2_-weighted images (T_2_WI) and hypointensity on T_1_-weighted images (T_1_WI). The presence of internal hemorrhage within the mass was suggested by hyperintensity on T_1_WI and hypointensity on T_2_WI. Diffusion-weighted imaging (DWI) showed restricted diffusion within the lesion. Following the administration of Gd-DTPA, the tumor exhibited heterogeneous enhancement during the arterial phase, with gradual enhancement observed in the portal and delayed phases.

The patient was transferred to our hospital for further evaluation and treatment. Upon physical examination, the findings were predominantly unremarkable, except for mild tenderness in the right upper quadrant. The patient’s medical history is notable for a number of chronic conditions: chronic hepatitis B, which has been present for over 20 years, type 2 diabetes mellitus for the past 12 years, and a historical episode of tuberculosis that was effectively treated and resolved 30 years prior. No other significant personal or family medical histories were reported. Laboratory tests indicated a slight decrease in serum albumin level, while other liver function indices were within normal limits. Viral markers of hepatitis B were positive, including hepatitis B surface antigen (HBsAg), antibody to hepatitis B e (anti-HBe), and antibody to hepatitis B core (anti-HBc). The antibody to hepatitis B surface (anti-HBs) and hepatitis B e antigen (HBeAg) were negative. The serum levels of tumor markers, including alpha-fetoprotein (AFP), carcinoembryonic antigen (CEA), and CA 19-9, were within the normal range.

**Figure 2:**
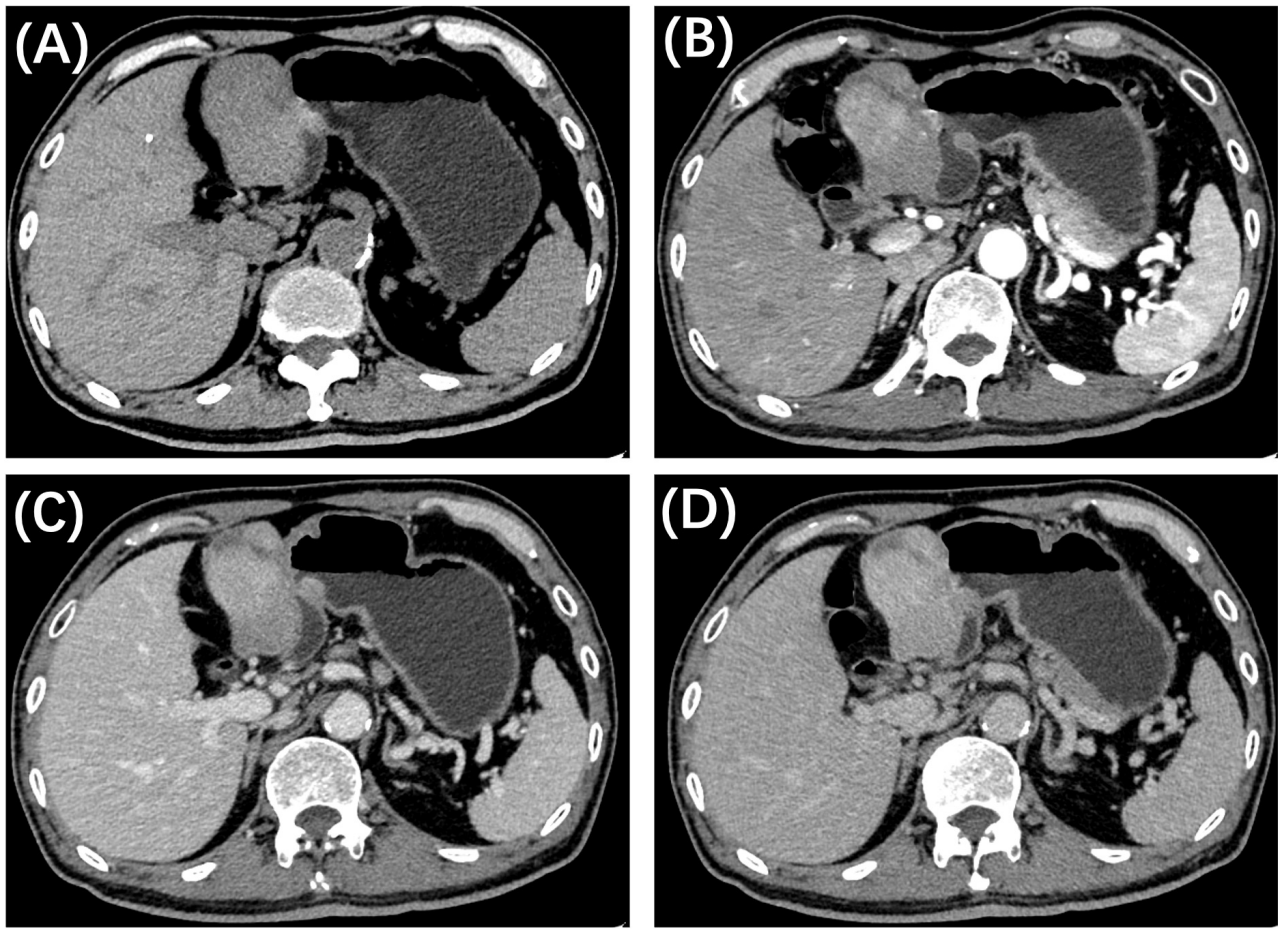
CT imaging of the present patient with primary hepatic leiomyosarcoma. **(A)** Axial precontrast CT reveals a well-defined, iso-dense mass in the left lobe of the liver, featuring a large exophytic component. Contrast-enhanced CT demonstrates uneven mild to moderate enhancement in the arterial phase **(B)**, with gradual enhancement observed during the portal **(C)** and delayed **(D)** phases.

Further evaluation was conducted through pre- and post-contrast computed tomography (CT, **Figure 2**) of the chest, abdomen, and pelvis. A well-defined, heterogeneous mass with hypo- and iso-density was identified in the hepatogastric space, adjacent to the left branch of the portal vein and the ligamentum teres hepatis, measuring approximately 6.8 cm × 4.5 cm × 5.8 cm. Mild to moderate uneven enhancement was observed during the arterial phase, with gradual enhancement during the portal and equilibrium phases. Feeding arteries were noted to originate from the left hepatic artery, suggesting that the large exophytic mass originated from segment IV and III of the liver. No evidence of hepatic cirrhosis or other clinically significant lesions was identified. Given the imaging features and the patient’s history of hepatitis B, a preliminary diagnosis of hepatocellular carcinoma (HCC) with atypical imaging appearances was established. A laparoscopic left hemi-hepatectomy was subsequently performed, and the patient had a smooth postoperative course. He was discharged 7 days following the surgery, marking a total hospital stay of 14 days.

Gross pathological examination demonstrated a gray-white tumor mass, measuring 6×5×4 cm in size. The cut surface exhibited as a grayish-white hue with scattered areas of hemorrhage and necrosis. The margin was tumor-free, and there was no evidence of lymphovascular or perineural invasion.

**Figure 3:**
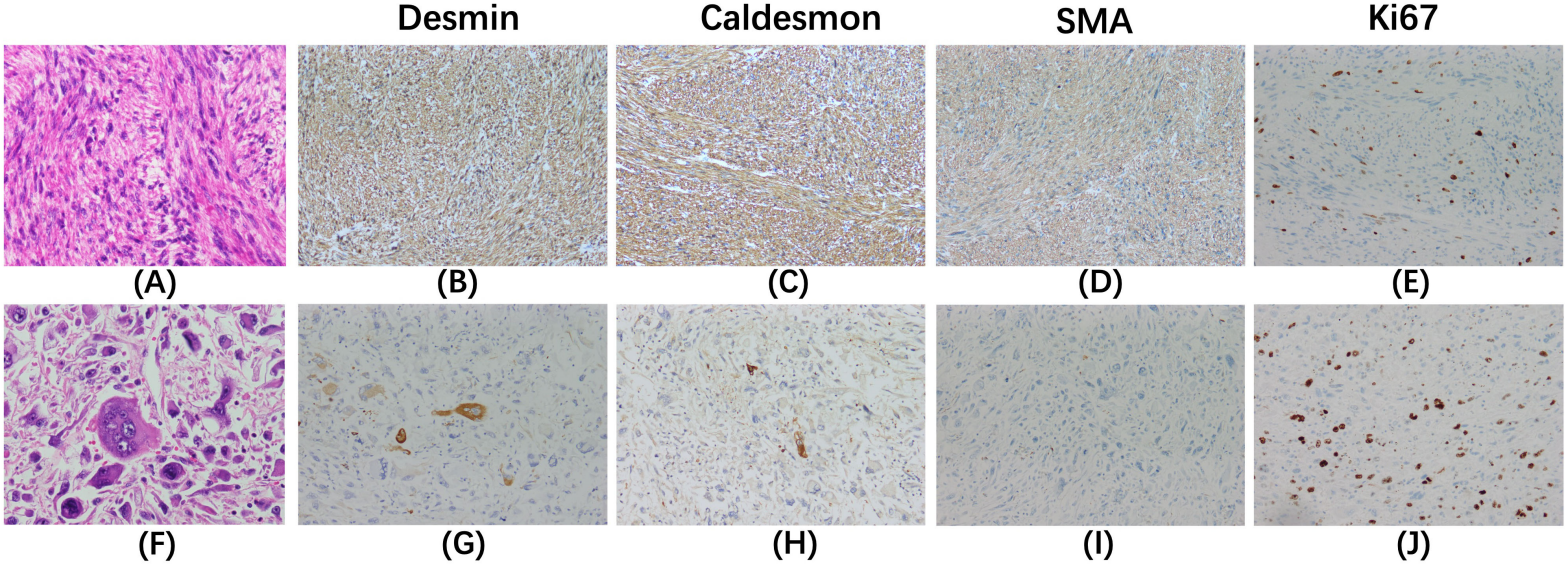
Hematoxylin-eosin staining (×400) and immunohistochemical staining (×200) are shown for the differentiated components **(A–E)** and the dedifferentiated components **(F–J)**. In the differentiated components **(A)**, cells with moderate nuclear atypia are densely packed in a fascicular and interwoven pattern. In contrast, the dedifferentiated components **(F)** display cells that are irregular or epithelioid, with marked nuclear atypia and a high mitotic rate. The differentiated area exhibits strong positivity for desmin **(B)**, caldesmon **(C)**, and smooth muscle actin (SMA, **D**). The dedifferentiated region shows patchy positivity for desmin **(G)** and caldesmon **(H)**, and is negative for SMA **(I)**. The Ki-67 index is significantly elevated in both the differentiated **(E)** and dedifferentiated components **(J)**.

Microscopic examination (**Figure 3**) revealed that the tumor was composed of spindle-shaped cells and had two discrete areas. One area was predominantly composed of ‘differentiated’ cells with moderate nuclear atypia, densely packed in a fascicular and interwoven pattern. These cells exhibited strong positivity for smooth muscle markers: desmin (3+), caldesmon (3+), and smooth muscle actin (3+). The other area was predominantly composed of ‘dedifferentiated’ cells with marked nuclear atypia and a high mitotic rate (approximately 40 per 5 mm²). These cells displayed irregular or epithelioid morphology alongside necrosis, and showed patchy positivity for desmin and caldesmon, and were negative for smooth muscle actin. The Ki-67 proliferation index was significantly elevated in both differentiated and dedifferentiated components. A final diagnosis of leiomyosarcoma was confirmed by a union discussion between the departments of Pathology of our hospital and another institution. The tumor was classified as grade 3 according to the Federation Nationale des Centres de Lutte Contre le Cancer (FNCLCC) grading system.

After the surgical procedure, the patient was informed about the potential benefits of adjuvant chemotherapy. Despite these considerations, the patient declined this treatment option and opted for regular follow-ups, which included periodic imaging and blood tests. After approximately 22 months of post-surgical monitoring, no local recurrence or metastasis was detected. Therefore, the diagnosis of primary hepatic leiomyosarcoma was comprehensive to made.

## 3 Discussion

Primary hepatic sarcomas account for less than 1% of all malignant hepatic tumors, with PHLS being even rarer, making up only 14-29% of all primary hepatic sarcomas (2–4). PHLS is believed to originate from smooth muscle cells within intrahepatic vessels, bile ducts, or ligaments (3). Given the scarcity of valuable data on clinical and imaging features, coupled with the tumor’s rarity, PHLS is frequently misdiagnosed as HCC or other conditions, such as cholangiocarcinoma, hepatocellular adenoma, focal nodular hyperplasia, abscesses, and hydatid disease (5–9).

The preoperative diagnosis of hepatic leiomyosarcoma is essential due to its metastatic potential, which necessitates a thorough diagnostic workup to exclude other primary sites (10). Such precise diagnosis is vital for devising appropriate treatment strategies, including the consideration of neoadjuvant therapy and the planning of intricate surgeries with broader margins to accommodate the aggressive characteristics of PHLS (11). Correct identification of PHLS is also essential to prevent the administration of inappropriate treatments that are typically prescribed for more prevalent liver tumors, such as HCC.

Therefore, we present this case of PHLS and review the existing literature to raise awareness of this uncommon malignant tumor. Our aim is to contribute to the clinical and radiological understanding of PHLS, ultimately improving patient management and outcomes.

### 3.1 Literature review

Case reports or case series of PHLS published in the English language from January 1, 2000, to December 31, 2023, were retrieved from PubMed and Google Scholar. After detailed screening of each article, 29 publications with 30 cases regarding the imaging features of PHLS were included in this study. The clinical manifestations and imaging findings of these 30 reported cases, along with the findings from the current case, are summarized in **Tables 1** and **2**, respectively.

**Table 1:** Clinical findings of PHLS from the literature and the present patient.

References	Case	Age	Sex	Symptoms	Tumor Markers			Past medical history	Treatment	Outcome
					AFP	CA19-9	CEA			
Ghosh, R. 2023 (5)	1	62	F	right upper abdomen pain	NM	NA	NA	unremarkable	right hepatectomy with cholecystectomy	NA
Ahmed, H. 2022 (3)	2	48	F	abdominal pain and weight loss	NA	NM	NA	diabetic, hypertensive, and morbidly obese	formal extended left hepatectomy	Alive and well at 6-month
Vella, S. 2020 (22)	3	77	F	vague, intermittent epigastric pain	NM	NA	NM	hypertension and cholecystectomy	laparoscopic right hepatectomy with resection of the lower root of the middle hepatic vein	Alive and well at 8-month
Kazawa, N. 2020 (17)	4	86	F	abdominal pain	NM	NM	NM	hypertension	NA	NA
Esposito, F. 2020 (11)	5	78	M	incidentally discovered during cardiac US	NM	NM	NM	NA	laparoscopic left hepatectomy	Alive with no recurrence at 18-month
	6	53	M	abdominal pain	NM	NM	NM	NA	Right extended hepatectomy	Recurrence at 7-month Died of recurrence at 14-month
Zhu, K.L. 2019 (23)	7	68	F	right upper quadrant pain and weight loss.	NM	NM	NA	NA	transcatheter arterial chemoembolization	no progressive enlargement of the tumor or distal metastasis at 82-month
Mitra, S. 2018 (21)	8	45	F	dyspepsia and weight loss	NM	mildly elevated	NM	NA	chemotherapy	Alive and well at 5-month
Liu, W. 2018 (6)	9	38	F	right upper abdominal pain and fever	NM	NM	NM	NA	expanded right hemi-hepatectomy chemotherapy	Lung metastasis at 25-month Liver recurrence at 39-month Then lost to follow-up
Feretis, T. 2018 (24)	10	68	F	right upper quadrant heaviness and discomfort	NM	NM	NM	chronic hepatitis B, type 2 diabetes mellitus and cholelithiasis	resection chemotherapy	recurrence at 18-month Died at 37-month
Xie, P. 2017 (25)	11	10	girl	jaundice	NA	NA	NA	NA	chemotherapy liver transplant	NA
Shera, I.2017 (26)	12	72	F	abdominal pain and swelling in the epigastrium and right hypochondrium, generalized weakness, loss of appetite and weight loss	NM	NM	NM	unremarkable.	chemotherapy	NA
Iida, T. 2017 (27)	13	63	F	general fatigue and fever	NM	NM	NM	autosomal dominant polycystic kidney disease	resected a part of the tumor	Died in a short time after the operation
Giakoustidis, D. 2017 (28)	14	69	F	abdominal pain	NM	NM	NM	unremarkable.	portal vein embolization resection of segments IVA, V, VI, and part of VII	Recurrence at 6 months Died of recurrence at 12-month
Gupta, S. 2016 (29)	15	45	F	loss of appetite, abdominal distension and bilateral lower limb swelling	NM	NM	NM	unremarkable.	upfront neoadjuvant chemotherapy	remained unresectable but stable After completing 6 cycles of NACT on regular follow up
Lv, W.F. 2015 (16)	16	42	M	abdominal pain, marasmus and weakness	NM	NM	NM	unremarkable.	NA	died of liver failure at 384-day
Metta, H. 2014 (12)	17	38	M	persistent fever, diarrhea, and productive cough	NM	NA	NA	human immunodeficiency virus (HIV) infection	Extended left hepatectomy	Alive with no recurrence at 1-year
Majumder, S. 2014 (30)	18	42	M	right upper quadrant abdominal pain and nausea	NA	NA	NA	NA	segment V hepatectomy with adhesiolysis and evacuation of the hematoma.	Alive with no recurrence at 3 -month
Tsai, P.S. 2013 (31)	19	5-month	girl	fever, nausea, vomiting, and poor appetite	NM	NA	NA	NA	chemotherapy Exploratory laparotomy with partial hepatectomy	Alive with no recurrence at 4-year
Chelimilla, H. 2013 (13)	20	54	M	intractable hiccups, low grade fever with poor oral intake and weight loss	NM	NA	NM	HIV/AIDS, seizure disorder, and hypertension	conservative management	NA
Takehara, K. 2012 (32)	21	59	M	demonstrated fecal occult blood and multiple liver tumors in a comprehensive medical examination	NM	NM	Slightly elevated	hypertension and hyperuricemia	chemotherapy + curative hepatectomy	Alive with no recurrence at 16-month
Shivathirthan, N. 2011 (33)	22	67	M	abdominal pain	NM	NM	NM	unremarkable	Extended left hepatectomy with extension onto the dorsal part of S8 preserving the MHV with partial resection of S6	Alive with no recurrence at 9-month
Morris, C.J. 2010 (8)	23	78	F	right upper quadrant abdominal pain, weight loss.	NA	NA	NA	NA	nonsurgical candidate	NA
Liang, X. 2010 (34)	24	44	F	anorexia and right upper quadrant pain	NM	NM	NM	hepatitis B infection	orthotopic liver transplantation	Metastasis at 14-month Died at 34-month
Surendrababu, N.R. 2006 (35)	25	1	boy	fever, right upper quadrant pain and vomiting	NM	NA	NM	NA	NA	NA
El Mesbahi, O. 2006 (36)	26	56	F	right sided abdominal pain, weight loss and fever	NM	NA	NM	hysterectomy for fibroma, diabetes and hypertension,	right hepatectomy and right hepatic lymphadenectomy with chemotherapy	alive at 18-month
Rokutanda, N. 2005 (37)	27	64	M	right upper abdominal pain	NM	NM	NM	NA	left hepatic lobectomy and cholecystectomy percutaneous radiofrequency ablation	Alive with no recurrence at 20-month
Kanazawa, N. 2002 (38)	28	31	M	discovered during routine health check	NM	NM	NA	unremarkable	left lateral segmentectomy	NA
Fujita, H. 2002 (15)	29	33	F	unexpectedly discovered on an abdominal US scan	NM	NA	NM	renal transplant recipients, right breast cancer	posterior segmentectomy	Alive with no recurrence at 2-year
Tsuji, M. 2000 (39)	30	68	M	right- sided abdominal distension and pain, weight loss	elevated	elevated	NM	hepatitis C virus-related liver cirrhosis	NA	died of rupture of the tumor 3-month after admission
Ours	31	66	M	abdominal distension	NM	NM	NM	chronic hepatitis B,	left hemi-hepatectomy	Alive and well at 22-month

F, female; M, male; NA, not available; NM, normal; AFP, alpha-fetoprotein; CEA, carcinoembryonic antigen.

**Table 2:** Imaging findings of PHLS from the literature and the present patient.

References	Case	Size (mm)	Location	Margin	US findings	CT		MR			
						Density	Enhancement	T_1_WI	T_2_WI	DWI	Enhancement
Ghosh, R. 2023 (5)	1	124	right lobe	well‐defined	a well‐defined heterogeneous hypoechoic mass	hypo-	AP: heterogeneous enhancement VP: persistent enhancement	NA	hyper-	NA	NA
Ahmed, H. 2022 (3)	2	99	left lobe	ill-defined	NA	NA	AP: heterogeneous contrast-enhancing and hyper vascular DP: partial wash-out	NA	NA	NA	NA
Vella, S. 2020 (22)	3	198	right lobe	NA	NA	Iso-& hypo-	NA	heterogeneous hypo-	slight hyperintensity with central heterogeneas hyper- & hypo-intensity	restricted diffusion	AP-PVP: peripheral gradual enhancement.
Kazawa, N. 2020 (17)	4	40	caudate lobe	NA	a para-caval hypoechoic mass	iso-hypo-	AP-PVP: gradually enhanced	hypo-	heterogeneous hyper-	heterogeneously high & low-	gradual mild enhancement
Esposito, F. 2020 (11)	5	57	left lobe	well-defined	NA	heterogeneous hypo-	mild and mostly peripheral wash-in and no wash-out	heterogeneous hypo-	hyper-	NA	discretely hypervascular
	6	290	right lobe	NA	NA	heterogeneous hypo-	late AP: peripheral enhancement	hypo-	hyper-	NA	AP-PVP: peripheral gradual enhancement
Zhu, K.L. 2019 (23)	7	100	right lobe	NA	a mixed echoic mass	inhomogeneous	mild delayed enhancement with central necrosis	NA	NA	NA	NA
Mitra, S. 2018 (21)	8	113	segment IV, V and VI	well-defined	a large heteroechoic lesion with cystic areas	heterogeneous hypo-	AP: heterogeneously enhancement and hypervascularity PVP: washout	hypo-	mild hyper-	mild diffusion restriction	gradual enhancement
Liu, W. 2018 (6)	9	136	right lobe	NA	a huge mass, which was interpreted as an abscess	heterogeneous hypo-	AP: enhancement of the wall with multiple tortuous vessels PVP&DP: wash-out	NA	NA	NA	NA
Feretis, T. 2018 (24)	10	130	left lobe segments III and IVB	irregular margin	a hepatic mass located in the left lobe of the liver	heterogeneous	NA	heterogeneous hypo-	heterogeneous hyper-	NA	NA
Xie, P. 2017 (25)	11	70	hepatic hilum	well-defined	a hepatic mass	NA	NA	hypo-	heterogeneous hyper-	restricted diffusion	NA
Shera, I.2017 (26)	12	NA	segment 4 and 3 of left lobe	NA	NA	heterogeneous	AP: early enhancement with interspersed non enhancing areas PVP: isointense to the liver parenchyma	NA	NA	NA	NA
Iida, T. 2017 (27)	13	100	left lobe	NA	NA	iso-	peripheral enhancement	slightly hypo-	slightly hyper-	NA	NA
Giakoustidis, D. 2017 (28)	14	140	segments IVA, V, VI and part of VII	NA	NA	hypo-	AP: heterogeneous enhancement PVP: delayed washout	NA	NA	NA	NA
Gupta, S. 2016 (29)	15	119	segment VII and VIII	well-defined	a hypoechoic mass	hypo-	AP: heterogeneous enhancement PVP: delayed washout	NA	NA	NA	NA
Lv, W.F. 2015 (16)	16	91	caudate lobe and the left lobe	well-defined	NA	slight hypo-	NA	slightly heterogeneous hypo-	heterogeneous hyper-	hyper-	AP&VP: no evident enhancement DP(5-min): marked enhancement
Metta, H. 2014 (12)	17	90	left lobe	well-defined	a hypoechoic mass in the left liver lobe, which increased by 30 mm over a 3-month period.	heterogeneous	heterogeneous enhancement	hypo-	hyper-	NA	irregular peripheral enhancement
Majumder, S. 2014 (30)	18	137	right lobe	well-defined	NA	heterogeneous	NA	centrally increased signal consistent with internal hemorrhage	hypointense rim compatible with a capsule or pseudocapsule	NA	peripheral enhancement
Tsai, P.S. 2013 (31)	19	65	right lobe	partially demarcated	a huge heteroechogenic mass	heterogeneous hypo-	enhanced heterogeneously and intensely	NA	NA	NA	NA
Chelimilla, H. 2013 (13)	20	35	right lobe	NA	NA	hypo-	rim enhancement	NA	NA	NA	NA
Takehara, K. 2012 (32)	21	42	left lobe	well-defined	hypo- or isoechoic heterogeneous masses without halos	hypo- or iso-	early enhancement and delayed washout or gradual enhancement	hypo-	hyper-	hyper-	delayed washout or gradual enhancement
Shivathirthan, N. 2011 (33)	22	170	left lobe and segment 8	NA	a hypoechoic mass	hypo-	AP: heterogenous enhancement PVP: delayed washout	NA	NA	NA	NA
Morris, C.J. 2010 (8)	23	174	right and left lobes	NA	NA	NA	NA	heterogeneous hypo-	heterogeneous hyper-	NA	peripheral enhancement
Liang, X. 2010 (34)	24	NA	right lobe	well-defined	a heterogeneous mass	hypo-	predominantly peripheral enhancement	NA	NA	NA	NA
Surendrababu, N.R. 2006 (35)	25	NA	right lobe		a large cystic lesion with multiple thick septations and few internal echoes, with no color flow on Doppler	hypo-	multiple enhanced septations	NA	NA	NA	NA
El Mesbahi, O. 2006 (36)	26	230	right lobe		NA	heterogeneous hypo-	NA	hypo-	hyper-	NA	peripheral enhancement
Rokutanda, N. 2005 (37)	27	140	left lobe	well-defined	a large cystic mass with a mixed echoic wall, including a hypoechoic internal lesion	heterogeneous hypo-	peripheral enhancement	hypo-	hyper-	NA	peripheral enhancement
Kanazawa, N. 2002 (38)	28	350	left lobe	NA	a low echoic mass with a clearly defined boundary	hypo-	peripheral enhancement	hypo-	peripheral isointensity with central heperintensity	NA	peripheral enhancement
Fujita, H. 2002 (15)	29	50	right lobe	well- defined	a large heterogeneous lesion	heterogeneous hypo-	NA	hypo-	hyper-	NA	NA
Tsuji, M. 2000 (39)	30	140	right lobe	well-defined	NA	hypo-	peripheral enhancement	hypo-	irregular hyper-	NA	peripheral enhancement
Ours	31	68	left lobe	well-defined	a hypoechoic mass in the hepato-gastric space	heterogeneous hypo- and iso-density	AP: mild to moderate enhancement PVP&DP: gradual enhancement	hypointensity with internal hemorrhage	heterogeneous hyperintensity with hypointense area	restricted diffusion	AP: heterogeneous enhancement PVP&DP: gradual enhancement

NA, not available; US, ultrasound; CT, computed tomography; MR, magnetic resonance; T1WI, T1-weighted images; T2WI, T2-weighted images; DWI, Diffusion-weighted imaging; AP, arterial phase; PVP, portal venous phase; DP, delayed phase.

### 3.2 Clinical manifestations of PHLS

The patients’ ages ranged from 5 months to 86 years, with a mean age of 52.4 years and three patients being under 18 years old. The prevalence was slightly higher among female patients, reflected in a female-to-male ratio of 1.4:1. The most common symptom was right upper quadrant abdominal pain. Other clinical presentations included abdominal distension, nausea, jaundice, weight loss, and a palpable abdominal mass, while some patients were incidentally discovered without apparent symptoms. Tumor marker levels, including AFP, CEA, and CA19-9, were typically within the normal range. Among the 30 reported cases, serum CA19-9 levels were slightly elevated in two patients, and serum AFP and CEA levels were each slightly increased in one patient. The current patient, an elderly male, presented with the clinical symptom of abdominal distension and had normal levels of tumor markers, a presentation similar to that of other abdominal malignancies and lacking specificity.

PHLS has been reported to be closely associated with immunocompromised states, including AIDS (12, 13), the post-renal transplant period (14, 15), and radiochemotherapy for Hodgkin’s lymphoma (9). This association is postulated to occur due to the uninhibited effects of the Epstein-Barr virus (EBV) on smooth muscle proliferation (12, 13). However, many patients diagnosed with PHLS were immunocompetent. Among the 31 patients, 3 (3/31, 9.6%) were immunodeficient, 4 (4/31, 12.9%) had a history of hepatitis B or C, with or without liver cirrhotic, and the remaining 24 (24/31, 77.4%) had no identifiable predisposing factors. Therefore, the etiology of PHLS remains unclear and requires further elucidation.

### 3.3 Imaging findings of PHLS

The non-specific imaging features of PHLS present a challenge for preoperative diagnosis. Conventional US was performed in 18 of the 31 patients, typically revealing a hypoechoic mass. CT scans were conducted on 29 of the 31 patients, with most showing well-defined hypo- or iso-dense masses that included heterogeneous areas indicative of necrosis or hemorrhage. MR imaging was performed in 20 of the 31 patients, showing hyperintensity on T_2_WI and hypointensity on T_1_WI. It may also exhibit internal heterogeneity, suggesting the presence of intratumoral hemorrhage or necrosis. The enhancement patterns of PHLS are comparable on both CT and MRI scans. Among the 31 cases, 23 patients received contrast-enhanced CT scans, and 15 received contrast-enhanced MR scans. A substantial proportion of these cases demonstrated peripheral, heterogeneous enhancement during the arterial phase, with persistent or gradual enhancement observed in the portal venous or delayed phases. Lv WF (16) and Kazawa N (17) have suggested that the gradual enhancement observed during the delayed phase on contrast-enhanced MR images may be a distinctive feature of PHLS. However, certain other hepatic tumors, including intrahepatic cholangiocarcinoma, atypical HCC, squamous cell carcinoma, and other sarcomas, frequently show delayed enhancement (18–20). Therefore, in our opinion, this feature could be a significant sign for the differential diagnosis of PHLS from typical HCC, but the diagnostic value for PHLS requires further study. In addition, some cases of PHLS reported in the literature exhibited the enhancement pattern described as “fast-in and fast-out” (6, 21), which is characteristic of the typical enhancement pattern seen in HCC. Diffusion-weighted imaging was performed in 7 of the 31 patients, revealing restricted diffusion in all cases.

In the present case, contrast-enhanced CT and MR imaging indicated a probable malignant hepatic lesion. Considering the patient’s history of hepatitis B, the initial misdiagnosis by radiologists and clinicians was HCC. However, with normal AFP levels and the scans showing gradual enhancement—features not aligning with HCC —the possibility of other malignant tumors necessitates consideration.

### 3.4 Imaging findings with pathologic correlation

A definitive diagnosis of PHLS relies solely on histological and immunohistological examinations. Characteristic histological features include intersecting bundles of spindle-shaped cells with deeply eosinophilic cytoplasm and hyperchromatic nuclei. Immunostaining is positive for SMA and desmin, which are markers indicative of smooth muscle differentiation. A negative reaction for CD34, CD117, DOG1, cytokeratins, neuron-specific enolase (NSE), S-100 protein, and alpha-fetoprotein aids in ruling out other differential diagnosess (3, 16, 21).

MRI is considered the most useful modality for characterizing liver masses due to its superior soft-tissue contrast resolution. In the present case, MR images revealed two distinct areas within the tumor, each exhibiting different imaging characteristics that correspond to the underlying pathological changes of PHLS. The area adjacent to the liver showed mixed high and low signals on T_2_WI and T_1_WI, corresponding to the undifferentiated region with necrosis and hemorrhage identified in pathological examination. The solid component of this area demonstrated significant hyperintensity on T_2_WI and a gradual enhancement pattern on dynamic contrast-enhanced images. In contrast, the area more distant from the liver displayed homogeneously mild hyperintensity on T_2_WI and marked gradual enhancement on dynamic contrast-enhanced images, correlating with the differentiated region composed of densely packed spindle-shaped cells as observed in pathological examination.

## 4 Conclusion

In conclusion, we report a case of PHLS, which is rare and lacks characteristic clinical and imaging manifestations. Based on the current patient and previously published data, PHLS often presents as a well-defined, heterogeneously hypo- or iso-dense mass on CT, with slightly prolonged T_2_ signal on MRI, and shows gradual enhancement during dynamic contrast-enhanced imaging. Although a definitive diagnosis of PHLS requires pathological examination, differentiation should be made once the aforementioned imaging features are present.

## Data Availability

The original contributions presented in the study are included in the article/supplementary material. Further inquiries can be directed to the corresponding author.
